# Evaluation of the Quality of Artemisinin-Based Antimalarial Medicines Distributed in Ghana and Togo

**DOI:** 10.1155/2014/806416

**Published:** 2014-10-27

**Authors:** Dorcas Osei-Safo, Amegnona Agbonon, Daniel Yeboah Konadu, Jerry Joe Ebow Kingsley Harrison, Mamadou Edoh, Andrew Gordon, Messanvi Gbeassor, Ivan Addae-Mensah

**Affiliations:** ^1^Department of Chemistry, University of Ghana, Legon, Accra, Ghana; ^2^Laboratory of Physiology/Pharmacology of Natural Substances, Faculty of Science, University of Lome, Togo; ^3^Department of Science Laboratory Technology, Accra Polytechnic, Ghana

## Abstract

This study, conducted as part of our overall goal of regular pharmacovigilance of antimalarial medicines, reports on the quality of 132 artemisinin-based antimalarial medicines distributed in Ghana and Togo. Three methods were employed in the quality evaluation—basic (colorimetric) tests for establishing the identity of the requisite active pharmaceutical ingredients (APIs), semi-quantitative TLC assay for the identification and estimation of API content, and HPLC assay for a more accurate quantification of API content. From the basic tests, only one sample totally lacked API. The HPLC assay, however, showed that 83.7% of the ACTs and 57.9% of the artemisinin-based monotherapies failed to comply with international pharmacopoeia requirements due to insufficient API content. In most of the ACTs, the artemisinin component was usually the insufficient API. Generally, there was a good correlation between the HPLC and SQ-TLC assays. The overall failure rates for both locally manufactured (77.3%) and imported medicines (77.5%) were comparable. Similarly the unregistered medicines recorded a slightly higher overall failure rate (84.7%) than registered medicines (70.8%). Only two instances of possible cross-border exchange of medicines were observed and there was little difference between the medicine quality of collections from border towns and those from inland parts of both countries.

## 1. Introduction

Artemisinin-based combination therapies (ACTs) which are currently the World Health Organization (WHO) recommended first-line treatment for uncomplicated malaria in endemic areas are under serious threat of resistance due to several reasons. The relatively high cost of these antimalarials has made their manufacture a lucrative venture for pharmaceutical industries; a situation that has led to the proliferation of diverse brands on the market. This has led some unscrupulous people to indulge in the manufacture of substandard and falsified brands [1–14]. The WHO acknowledges the difficulty that this situation presents to the quality assurance of antimalarials on the market, especially in developing countries where enforcement of laws regarding manufacture, importation, and distribution of medicines is relatively lax. Ghana and Togo have already adopted artemether/lumefantrine and artesunate/amodiaquine as their first-line treatment for* Plasmodium falciparum* malaria but according to the World Malaria Report 2013 these countries still present high endemicity with 100 percent of their populations living in high transmission areas [15].* P. falciparum* resistance has been confirmed in several parts of South East Asia, where the problem of counterfeit medicines is well-organised [1–3, 6, 7].

The manufacture, distribution, and use of poor quality medicines (degraded, substandard, and counterfeit) are major factors in the development of resistance. There has been considerable global controversy and tensions among public health stakeholders regarding the definitions of categories of poor quality medicines. The 1992 operational definition of counterfeit medicines adopted by the WHO and later revised by IMPACT in 2008 have been criticised for incorporating intellectual property and legal considerations, thus diverting attention from the core issues of safety, quality, and efficacy of medicines [16–20]. The problem led to the WHO 2010 proposal thatuntil consensus was reached, medical products produced or distributed with the intent of fraud could be described as “substandard/spurious/falsely labelled/falsified/counterfeit medical products (SSFFC)” [21]. However, this lumping of all poor quality medicines together has been described as creating a misleading impression that they all have similar deficiencies. New proposals suggest that substandard medicines should be separated from counterfeit products and that the term “counterfeit” should be excluded from the definitions for the purpose of international cooperation [18, 19]. Thus substandard medicines are defined as pharmaceutical products produced by legitimate manufacturers (originator and generic) which do not meet their quality standards and specifications [22–24]. Falsified medicines, like substandard ones, also do not meet quality specifications; the distinction is that there is a deliberate intent to breach regulatory requirements [18, 19, 25]. Both substandard and falsified medicines pose a serious threat to public health. Since the ACTs remain the most effective treatment for uncomplicated and* P. falciparum *malaria, it is extremely important to monitor the quality of our ACTs, as part of the measures to contain the spread of ACT-resistant parasites to the malaria-endemic African region.

Ghana has benefitted more than Togo from several WHO-sponsored and other studies carried out to evaluate the quality of ACTs distributed in the malaria-endemic regions of Africa [8, 11, 26, 27]. Notable among these is the 2011 report of a WHO survey of the quality of selected antimalarial medicines circulating in six countries of sub-Saharan Africa which showed that products from Nigeria had the highest failure rate (64%) followed by Ghana (39%) and then Cameroon (37%). Among ACT samples tested in Ghana, failures resulted from insufficient API, related substances test and tablet mass uniformity test, indicating inconsistencies in the implementation of GMP in both domestic and imported products. Although there was also a substantial amount of unregistered products especially among the ACTs, the nature of failure rates for registered and unregistered medicines was similar. Generally, the results of the survey were not considered representative enough to conclude on the quality of medicines in Ghana and further investigations were proposed. Nonetheless, it was recommended that the Food and Drugs Authority (FDA) should strengthen its regulatory and surveillance systems to minimize the presence of unregistered medicines. The relatively high number of manufacturers supplying either registered or unregistered products was considered a challenge to quality assurance of antimalarials in Ghana [12]. The global report on antimalarial medicine efficacy and medicine resistance (2000–2010) includes studies carried out in both Ghana and Togo. Whereas, in 2006, the treatment failure rate in Ghana was greater than the 10% threshold at which the WHO recommends an initiation of a change in medicines in national treatment policies, similar studies carried out in Togo around the same period gave treatment failure rates of 4.4% for artemether/lumefantrine and 6% for artesunate/amodiaquine [28]. These findings suggest that regular monitoring to assess the quality of antimalarial medicines circulating in Ghana and other countries in the subregion is crucial.

Our recent study which involved the validation and application of quality assurance methods to antimalarial medicines distributed in Accra, Ghana, identified substandard samples, with insufficient API [11]. In the current study, the sampling area extended outside Accra to cover other major cities and towns, with special interests in the border areas, since porous borders have the potential of serving as conduits of poor quality antimalarial medicines circulating within the subregion and beyond. Since Togo has not been included in many of the medicine quality surveys, this was a good opportunity to undertake a comprehensive quality evaluation of ACTs distributed in Togo. The major objective of the present study was to determine the quality of artemisinin-based antimalarial medicines available to the consumers in the two countries with respect to the International Pharmacopoeia (Ph. Int.) compliance of the API content.

## 2. Materials and Methods

### 2.1. Sampling

In both countries, the sampling design was mainly by convenience sampling since it did not follow a complete list of outlets in defined areas. However, sampling sites were chosen based on the level of economic activity (both high and low) and proximity to border towns. All brands of locally manufactured as well as imported artemisinin-based antimalarial medicines available to the consumer were collected. Since consumers usually buy from retail outlets (pharmacies and licensed chemical shops) more than from wholesalers, majority of the samples were collected from retail outlets. Another reason for purchasing most of the samples from retailers instead of wholesalers was to cut down on cost. For example, if three packets of a medicine containing ten tablets each were enough for chemical analysis, it was cheaper to purchase these three individual packets from a retailer than buy a whole box of the same medicine containing about twenty packets from a wholesaler. This strategy enabled us to buy more samples that belonged to different batches of the same medicine. The samplers constituted members of the research team and they posed as normal shoppers buying medication. When different and large quantities of antimalarials were being bought from the same seller it was sometimes necessary to explain that they were for research purposes. In such cases, some sellers sold out freely, possibly because they were doing good business, while others did so with some reservation.

**Figure 1:**
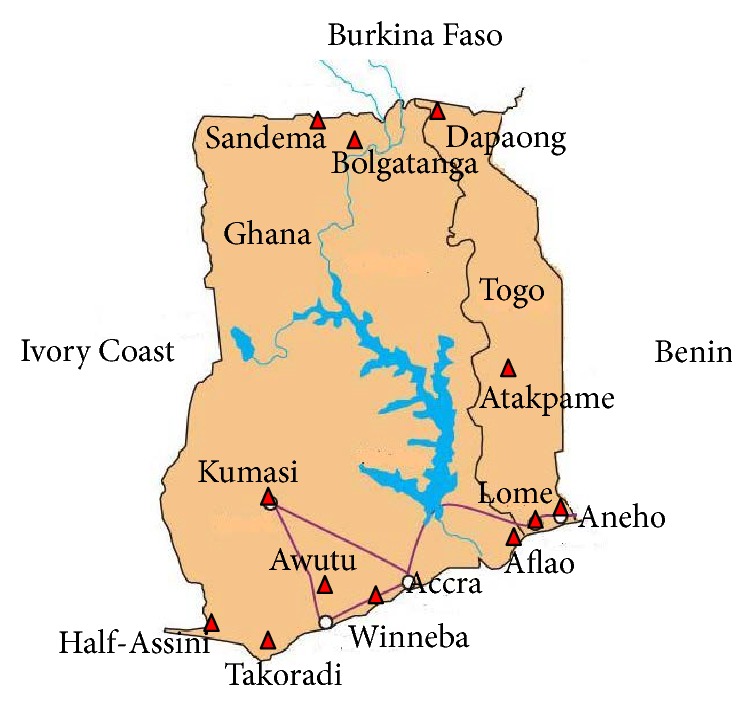
A map showing the sampling areas (red triangle) in the two countries.

Togo has 5 economic zones, namely, Maritime, Plateaux, Central, Kara, and Savanes Regions (Figure 1). The antimalarial medicines were collected between December 2010 and March 2011 from four sampling sites in three of the designated zones as follows: Maritime Region-Lomé (Zone 1) and Aného (Zone 2); Plateaux Region-Atakpame (Zone 3) and Savanes Region-Dapaong (Zone 4). They were purchased from both regular pharmacies and nonaccredited medicine stores in the market from Lomé area. It is important to mention that antimalarial medicines sold in regular pharmacies in the country come from 4 large commercial suppliers all located in Lomé: UNIPHART (Union des Pharmaciens du Togo), CAMEG (Centrale d'Achat des Médicaments Essentiels et Génériques), SOTOMED (Société Togolaise des Médicaments) and GT-PHARM (Groupement Togolais des Pharmaciens).

In Ghana, 6 sampling sites located in the coastal, the middle, and the savannah belts of the country were selected. A previous study on the quality of antimalarial medicines in Ghana focused on ACTs distributed in Accra (Zone 1); therefore the present study excluded Accra [11]. Takoradi (Zone 3) in the coastal belt and Kumasi (Zone 6) in the middle belt were chosen due to the high level of economic activity in these cities. Kumasi is the second largest city in Ghana while Takoradi is the third largest, but it also has the country's second largest seaport which handles most of the country's cocoa and rubber exports. In addition, it is the largest city in the country's oil producing region and also handles exports of minerals such as manganese and bauxite. Winneba and Awutu, two relatively small rural towns, were selected as Zone 2. Bolgatanga/Sandema (Zone 4) in the savannah belt, Aflao (Zone 5), and Half-Assini (Zone 7), also in the coastal belt, were selected based on their situation near the borders of the country. They are border towns with Burkina Faso, Togo, and La Cote d'Ivoire, respectively (Figure 1). All the samples were purchased between September 2010 and April 2011 and together with the Togo collection were analysed within the period of their shelf lives.

### 2.2. Quantities and Categories of Antimalarial Medicines

A total of 132 antimalarial medicines were collected from the two countries, 58 (43.9%) from Ghana and 74 (56.1%) from Togo. They comprised 90 (68.2%) artemisinin-based coformulated and artemisinin-based coblistered medicines (ACTs) and 42 (31.8%) artemisinin-based monotherapy formulations. Thirty nine of the samples contained artesunate, 14 of which were all oral monotherapy. None of the recommended parenteral artesunate for managing severe malaria was collected. A more in-depth survey may confirm the dearth or otherwise of these vital treatments in both countries. There were 81 samples containing artemether, out of which 22 were monotherapy formulations (injections and suppositories). The 12 dihydroartemisinin containing medicine formulations consisted of 6 monotherapy formulations.  Table 1 gives a breakdown of the antimalarial medicines obtained from both countries per the product type, outlet, and the zone of collection while Table 2 shows a further breakdown of the product type.

### 2.3. Manufacturing Source

The countries of origin of the samples show a huge reliance of the two countries on importation of medicines (80.3%). The majority of the samples (50.8%) were stated as manufactured in India, followed by 12.9% in Ghana. Nine (6.8%) samples were stated as originating from Togo while the rest were stated as manufactured in nine other countries: Morocco, Senegal, China, England, Germany, France, the United States, Vietnam, and Spain.

### 2.4. Reference Standards

WHO International Chemical Reference Substance (ICRS) for all samples analysed was obtained from the European Directorate for the Quality of Medicines and HealthCare (EDQM), Strasbourg, France.

### 2.5. Analytical Tools

#### 2.5.1. Visual Inspection

Prior to the more rigorous chemical assays, visual inspection of packaging and dosage form was employed as a quick means of checking the quality or otherwise of the samples. The packaging was checked for correct and legible labelling of active ingredients and strength, expiration date, batch number, manufacturer, and country of origin. The study did not go as far as forensic examination of trademarks, product designs, or holograms. The appearance of the samples was also examined for discolouration, chippings, or excessive powder. Registration verification with the national medicine regulatory authorities was also done.

#### 2.5.2. Basic (Colorimetric) Tests

Basic (colorimetric) tests, also known as simplified tests, provide simple and readily applicable methods for confirmation of the identity of active pharmaceutical ingredients (APIs). To ensure that all the samples contained the requisite API, each of them was evaluated using methods described in the WHO Basic Tests for pharmaceutical dosage forms [29] together with various WHO restricted documents that are prepared for incorporation into the International Pharmacopoeia. The details of the analysis are as previously published [11].

#### 2.5.3. Semiquantitative TLC (SQ-TLC) Assay

**Figure 2:**
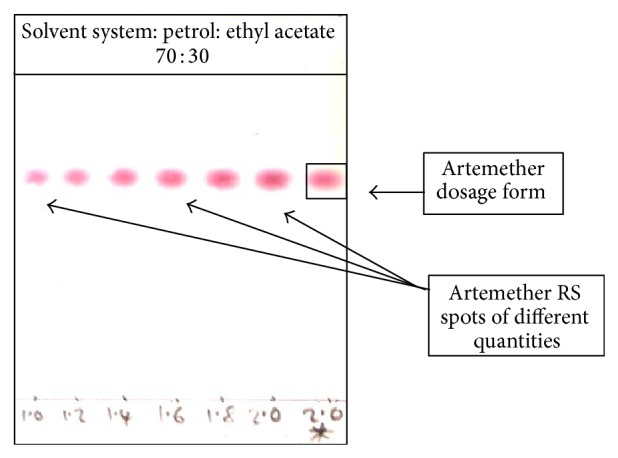
A sample of developed TLC plate of an artemether-containing medicine.

The previously described SQ-TLC protocol [11] was employed as a rapid, simple, and affordable quality monitoring tool to estimate the API content of the samples.  Figure 2 shows a chromatogram of an artemether-lumefantrine coformulated medicine assayed for artemether API content. Applied to the plate were 2.0 *μ*L of a 1 mg/mL solution of the medicine sample and varying volumes of 1 mg/mL solution of the RS (1.0–2.4 *μ*L). When the chromatogram was scanned and saved onto a computer, application of Microsoft Office Picture Manager in varying the intensity of the spots gave the following observations: the artemether API of the sample at 2.0 *μ*L^*^ started to fade from the TLC plate around the same time as the artemether RS spot at 1.6 *μ*L but faded completely before the artemether RS spot at 1.8 *μ*L. Since these volumes are equivalent to the corresponding quantities of the API in *μ*g, this observation implies that the actual amount of API contained in the sample is between 1.6 *μ*g and 1.8 *μ*g of pure API and not the expected 2.0 *μ*g. Since the label claim of the dosage form is 20 mg of artemether, the lower percentage limit is, 1.6/2.0 × 100 = 80%, equivalent to 16 mg per tablet of artemether, while the upper limit is 90%, equivalent to 18 mg per tablet of artemether. A range of 80–90% of artemether API is not compliant with the WHO international pharmacopoeia requirement which stipulates that each tablet must contain not less than 90% and not more than 110% of the amount of artemether stated on the label [30]. The SQ-TLC assay of the sample therefore suggests that this sample is noncompliant with respect to API content.

#### 2.5.4. HPLC Assay

To further confirm and validate the results of the SQ-TLC, all the medicine dosage forms were assayed using HPLC as a more accurate method of quantitative analysis. Calibration curves were prepared using the various reference standards (RS). The experimental details for preparation of solutions of the APIs in the dosage forms were previously indicated [11]. The area under the curve (AUC) for each of them was calculated from their respective chromatograms obtained from the assay. Six replicates were obtained for each API. The average AUC was then calculated and their concentrations determined from the calibration curves.

**Figure 3:**
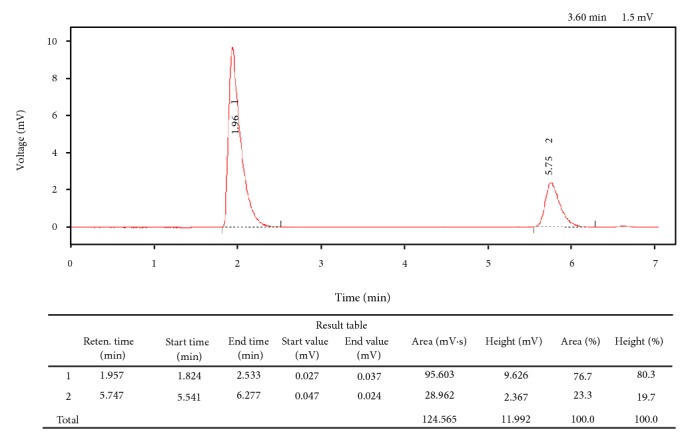
Chromatogram of a preparation containing 0.6 mg/mL artesunate and 1.8 mg/mL amodiaquine.

*Artesunate Single Component and Artesunate/Amodiaquine Coblistered and Artesunate-Amodiaquine Coformulated Medicines*. Although the Ph. Int. describes separate methods for the assay of artesunate and amodiaquine, there appears to be no pharmacopoeia method for the simultaneous assay of artesunate-amodiaquine coformulated medicines [31–33]. Thus slight modifications to the experimental conditions described by Gandhi et al. [34] were applied in the current study.  Figure 3 shows a chromatogram of a preparation containing 0.6 mg/mL artesunate and 1.8 mg/mL amodiaquine.

**Figure 4:**
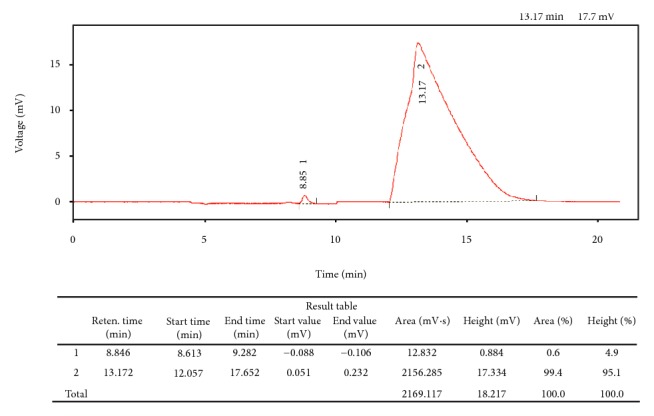
Chromatogram of a preparation containing 0.4 mg/mL artemether and 2.4 mg/mL lumefantrine.

*Artemether Single Component, Lumefantrine Single Component, and Artemether-Lumefantrine Coformulated Medicines*. In view of the large number of samples to be analysed and considering the long retention time for the Ph. Int. gradient method, (artemether: 19 minutes; lumefantrine: 34 minutes) [30], a modified method resulting in a shorter retention time was developed and used in the present study. Extraction of API from the medicine dosage forms was done with acetic acid followed by acetonitrile. While lumefantrine ionizes in acetic acid causing it to dissolve, artemether is highly soluble in acetonitrile. Therefore, acetic acid would dissolve lumefantrine while acetonitrile would dissolve artemether, causing both APIs to be soluble in the solvent mixture. To overcome the issue of low concentrations of artemether in the coformulated medicine (16.7%) coupled with its low molar absorptivity, higher concentrations of the medicines were prepared to enable the detection of artemether while at the same time being mindful of unnecessarily overloading the column with lumefantrine. The linearity of this intervention, when tested with the calibration curve, gave *r* ^2^ values of 0.995 for lumefantrine and 0.999 for artemether.  Figure 4 shows a chromatogram of a preparation containing about 0.4 mg/mL artemether and 2.4 mg/mL lumefantrine.

*Dihydroartemisinin Single Component and Dihydroartemisinin-Piperaquine Coformulated Medicines*. A modified version of the method described in the Ph. Int. was used in the HPLC assay of dihydroartemisinin dosage forms [33].

## 3. Results 

### 3.1. Reporting of Results

The findings of the study have been submitted to the funding body, West Africa Health Organisation (WAHO), and are also available in the students' thesis reports which are now in the public domain. No official report has, however, been made to our NMRAs, the companies, or the WHO Rapid Alert System.

### 3.2. Visual Inspection and Registration Verification

Visual inspection did not reveal any false-labelling; however, one oral artesunate monotherapy among the Ghana samples appeared slightly chipped and powdery. Overall, about 46% of the collected samples were not registered by either country's respective medicine regulatory authority. A greater proportion of the Ghanaian collection (79.3%) was unregistered while Togo had only 21.6% unregistered samples, suggesting a possible more rigorous enforcement of regulatory laws in Togo than in Ghana.

### 3.3. Basic Tests

Results of the basic tests indicated that with the exception of the slightly chipped and powdery oral artesunate monotherapy sample in the Ghana collection, which totally lacked the API, all the antimalarial medicines analysed contained the requisite APIs.

### 3.4. Semiquantitative TLC and HPLC Assays

Due to the large data generated from both assays, a selection of the results is presented in Table 3. Three categories of antimalarial medicines were identified based on the percentage or quantity of the API present. The first category had acceptable quantities of API and complied with Ph. Int. requirements (not less than 90% and not more than 110% of the amount of API stated by the label) [30]. This category was labeled compliant (C). The second category contained medicines which did not comply with Ph. Int. requirements because the quantities of their APIs were either below or above the pharmacopoeia limits. They were described as noncompliant (NC). However, some samples were identified to have recorded percentages that were marginally compliant with the upper and lower Ph. Int. requirements. Thus another category was created for them and labeled border-line compliant (BLC). For the SQ-TLC assay, samples which fell within ±5% of the upper and lower limits (i.e., 85% to 115%) were considered BLC while a margin of ±2% was set for the more accurate HPLC assay. Failure of a sample was based on the pharmacopoeia noncompliance of the API component of the medicine formulation. For the ACTs, a sample was considered to have failed if either both or one of the APIs did not meet pharmacopoeia specifications. Statistical analysis was carried out using ANOVA at 95% confidence level.

The SQ-TLC results obtained using the two different solvent systems described previously were comparable and therefore complemented each other. The results of the basic tests were largely corroborated by the SQ-TLC in that the visualizing agents used in developing the chromatograms gave characteristic colours indicative of the nature of a specific API present. Cobaltous nitrate saturated with sodium chloride was used for the first time as the visualizing agent in the SQ-TLC determination of amodiaquine and was found to be a better detection reagent than the I_2_/KI solution normally used for amodiaquine [35].

The only sample (3N_7_, Table 3) which failed the visual inspection of dosage form as well as the basic test was not detected by both SQ-TLC and HPLC, corroborating the previous observations. However, one artesunate monotherapy medicine from the Togolese collection which gave a positive basic test appeared differently on TLC in both the colour of the spot and *R*_*f*_ with respect to the RS. The API was not detected by HPLC either. Possibly, the component in this tablet purported to be artesunate was not artesunate.

### 3.5. Artesunate and Artesunate/Amodiaquine Samples

There were 39 artesunate-containing samples, 14 of which were all oral monotherapy. Thirty-seven of the artesunate-containing samples were tested and the results showed a 100% failure rate for the artesunate component by both SQ-TLC and HPLC analyses. Apart from two artesunate monotherapy formulations, one from each country, which did not contain any active ingredient, all the other samples contained insufficient quantities of the active ingredient as indicated by the manufacturers. ANOVA at 95% confident interval for artesunate-containing samples collected from both Ghana [*F*(2,39) = 0.028, *P* > 0.05] and Togo [*F*(2,46) = 0.431, *P* > 0.05] did not reveal a statistically reliable difference between the means of artesunate recovery using TLC solvent system one, TLC solvent system two, and the HPLC method. Analysis of the amodiaquine component of the medicines, however, indicated that 13 out of 14 of the Ghana collection met Ph. Int. requirements using both methods, while one sample failed by both SQ-TLC and HPLC analyses [*F*(2,42) = 0.016, *P* > 0.05]. All the 11 amodiaquine-containing medicines obtained from Togo passed the content requirement tests using both methods [*F*(2,42) = 0.001, *P* > 0.05].

### 3.6. Artemether and Artemether/Lumefantrine Samples

Thirty-two samples were collected in Ghana: one artemether injection and 31 artemether-lumefantrine ACTs. The injection passed both assays while, of the 30 out of the 31 ACTs analysed for artemether content, 22 samples (73.3%) and 25 samples (83.3%) failed using HPLC and SQ-TLC assays, respectively. ANOVA did not reveal a statistically reliable difference between the means of artemether recovery using TLC solvent system one, TLC solvent system two, and the HPLC method. [*F*(2,90) = 0.295, *P* > 0.05]. On the other hand, only one sample out of the 31ACTs failed the content requirements for lumefantrine by both SQ-TLC and HPLC analyses [*F*(2,90) = 0.275, *P* > 0.05]. Incidentally, the artemether component of this medicine passed HPLC analysis. Considering the quantities of both components, however, only 9 out of the 31 samples were fully compliant, comprising 8 coformulated medicines and the only artemether injection. The Togo samples comprised 4 artemether suppogels, 17 artemether injections, and 28 artemether-lumefantrine coformulated tablets. The assay for artemether content gave the following results: 14 artemether out of the 17 injections failed the SQ-TLC assay while the remaining 3 were border-line compliant; only 5 out of the 17 injections failed the HPLC assay-7 were fully compliant while 5 were border-line compliant. In the 28 ACTs, artemether content failed by 100% and 85.7% in SQ-TLC and HPLC assays, respectively. ANOVA, however, failed to reveal a statistically reliable difference between the means of artemether recovery using TLC solvent one system, TLC solvent two system, and the HPLC method. *F*(2,129) = 0.662, *P* > 0.05. The artemether suppogels could not be assayed due to interference by the excipient.

The assay for lumefantrine in the 28 artemether-lumefantrine coformulated tablets gave the following results: 100% failure by SQ-TLC; 21.4% failure, 28.6% marginally compliant, and 50% fully compliant by HPLC. ANOVA revealed a statistical reliable difference between the means of lumefantrine recovery using TLC solvent one system, TLC solvent two system, and the HPLC method [*F*(2,48) = 4.123, *P* < 0.05].

### 3.7. Dihydroartemisinin (Artenimol) Containing Samples

Twelve dihydroartemisinin-containing samples (6 ACTs and 6 monotherapy formulations) were collected from both countries. The Ghana collection comprised four dihydroartemisinin/piperaquine ACTs and five dihydroartemisinin monotherapy formulations while the Togo collection comprised two ACTs (dihydroartemisinin/piperaquine and dihydroartemisinin/sulfadoxine/pyrimethamine) and one monotherapy formulation. In each collection, the failure rates for the two dosage forms were similar and the SQ-TLC assay results were largely confirmed by the HPLC results. ANOVA showed no statistical difference between the means of sample recovery using TLC solvent one system, TLC solvent two system and the HPLC method. *F*(2,24) = 2.146, *P* > 0.05. For the Togo samples, while the co-formulated sample with sulfadoxine/pyrimethamine could not be assayed by HPLC due to interference by the sulfadoxine component, the SQ-TLC showed that the dihydroartemisinin API component was more than the manufacturer's label claim (110–120%) and hence noncompliant. The coformulated sample with piperaquine was similarly noncompliant (113–120%).

## 4. Discussion

Generally, the results of the SQ-TLC assay of the antimalarials were validated by the corresponding HPLC results. With exception of an artesunate single component copackaged amodiaquine, in which the amodiaquine component failed by SQ-TLC assay but passed the HPLC assay, SQ-TLC assay of all the artesunate-containing samples collected from both countries was confirmed by the HPLC results. A similar analogy can be made for the dihydroartemisinin samples. However, in the assay of artemether and lumefantrine, estimation of API content by SQ-TLC gave some results that were on the lower limit as compared with HPLC results (Tables 4 and 5). This has been attributed to failure to exhaustively extract the API in such situations prior to SQ-TLC analyses. Lumefantrine API assayed by SQ-TLC was noncompliant for all samples collected from Togo while only 5 samples failed in the HPLC assay. Thirteen samples were fully compliant while 8 were marginally compliant. This discrepancy has been traced to nonexhaustive extraction of the lumefantrine API by acetonitrile for SQ-TLC assay. For the HPLC assay, ethyl acetate was used as the solvent for extraction. It was also used in the extraction of the Ghana samples for both the SQ-TLC and HPLC assays and the results were comparable. In the case of the artemether injections which recorded better quality with respect to the HPLC assay, the liquid formulation may have favoured analysis by HPLC more than SQ-TLC.

Substandard medicines are defined as pharmaceutical products produced by legitimate manufacturers which do not meet their quality standards and specifications [22–24]. Falsified medicines like substandard ones also do meet quality specifications; the distinction is that there is a deliberate intent to breach regulatory requirements [18, 19, 25]. None of the sample packages was identified to be wrongly or falsely labelled and even for the two oral artesunate monotherapies whose API was not detected, it is difficult to determine this as an intentional breach of regulatory requirement. Thus the results suggest that the noncompliant samples are substandard. The increasing incidence of production and distribution of substandard medicines by genuine manufacturers operating legally in many developing countries poses a major health hazard and this places greater responsibility on national drug regulatory agencies to ensure the production of quality drugs for use in their countries.

The high failure rate especially of the ACTs was mostly due to insufficient quantities of the artemisinin component in the dosage forms. For instance all the artesunate/amodiaquine ACTs from both countries failed due to insufficient artesunate API. A possible implication of this is that, for artesunate/amodiaquine ACTs or monotherapy, it appears that manufacturers may be deliberately putting in smaller amounts of the more expensive artesunate component while the right amounts of the less expensive nonartemisinin components are kept at a minimum. Hence cures may be obtained even when the medicine used does not meet pharmacopoeia standards. It has been observed that treatment of malaria with artesunate/amodiaquine combination therapy has dramatically improved efficacy over amodiaquine alone [36–38]. Addition of artesunate to amodiaquine is also reported to reduce gametocytemia compared to amodiaquine monotherapy, thereby theoretically reducing transmission [37, 38]. The presence of insufficient quantities of artesunate API is therefore a cause for concern because administration of suboptimal doses of medicines to parasites even though in many cases reduces symptomatic episodes of the disease in patients leaves many uncleared parasites in the body of individuals. This has been a major intermediate in the development of resistance because upon reinfection, these parasites which have been exposed to subtherapeutic doses of the medicines have been shown to undergo mutations that make them tolerant to the medicines [39, 40]. Artemether and artemether/lumefantrine antimalarial medicines formed the bulk of the samples analysed (77, 18 of which were parenteral monotherapy). This is a clear indication of the widespread use of artemether/lumefantrine as the preferred therapeutic agent due to its tolerability. The results obtained by both SQ-TLC and HPLC analyses of the Ghana collection were not different from the trend observed in the analysis of the artesunate/amodiaquine samples. In most cases, while the artemether component was insufficient, the lumefantrine component was compliant. Failure in dihydroartemisinin API content was a result of the presence of either insufficient or overdose quantities. The problem with overdosing is that it puts patients at the risk of toxicity which is equally dangerous.

### 4.1. Quality of Antimalarial Medicines versus Manufacturing Source and Registration Status

Since the two countries rely heavily on importation of antimalarial medicines (80.3%) from different countries, and Ghana especially had significant quantities of unregistered medicines (76.3% unregistered for Ghana and 21.6% unregistered for Togo), a quality assessment of medicines from domestic and foreign sources as well as for registered and unregistered medicines was carried out. The data is presented in Table 6.

It was observed that the overall failure rates of the samples assayed were comparable irrespective of manufacturing source with 77.3% of domestic samples and 77.5% of imported samples failing. A country by country analysis, however, showed almost twice the failure rate in locally manufactured medicines in Ghana (92.3%) compared to Togo (55.6%). Failure rates in the imported samples were similar: 76.2% for Ghana and 78.3% for Togo. In the case of registration status, a greater percentage of registered medicines (83.8%) than unregistered medicines (79.1%) failed in Ghana. All the 16 unregistered Togo samples and 68% of the registered samples failed. The overall figures of 70.8% failure for registered medicines and 84.7% for unregistered medicines suggest that the registration status of a medicine does not necessarily translate into quality. These findings further suggest that the quality of the antimalarial medicines may have been compromised at the manufacturing stage rather than through the distribution chain since no decomposition products were observed during the assay by SQ-TLC and/or HPLC.

### 4.2. Cross-Border Activities

The porous nature of the West African borders makes cross-border exchange of goods including medicines relatively difficult to control. In order to determine possible cross-border transfer of antimalarials, and whether this had any significant impact on quality, collection and analysis of medicines from areas at and close to the borders of the two countries were carried out. The variation observed in the quality of the antimalarial medicines collected from border areas and inland cities was insignificant. The more significant observation confirming possible cross-border exchanges or distribution by the same importers was the occurrence of medicine samples of the same batch from the same manufacturer being found in the two countries. There were two sets of artemether-lumefantrine coformulated tablets from the two countries with the same batch number. There was no evidence of registration of these batches with the regulatory authorities in either country. In set one, both component APIs were compliant in the Ghana collection (5P_3_^f^), whereas insufficient quantities of artemether was found in the Togo collection (1PM2). In the second set, both API components in the Togo collection (2PM2) as well as artemether API in the Ghana collection (6P_4_^f^) were noncompliant. Samples 5P_3_^f^ and 1PM2 could easily have been exchanged in cross-border activity because they were collected in the border town of Aflao (Ghana) and Lome (Togo), respectively. On the other hand, samples 2PM2 and 6P_4_^f^ were collected at Aneho (eastern border of Togo) and Half Assini (western border of Ghana), respectively. A dihydroartemisinin sample (1QM6), collected from Lome, Togo, also belonged to the same batch as a Ghana (Half Assini) collection coded 6Q_1_. While sample 1QM6 gave an SQ-TLC range of 110–115% making it border-line compliant, the HPLC showed it was compliant, with a value of 106.77% of the manufacturer's label claim. On the other hand, sample 6Q_1_ failed both SQ-TLC (70–75%) and HPLC (80.75%) analyses, revealing the variation in composition of API even within same batches of the same medicine. These findings suggest the possible existence of extensive cross-border distribution of medicines between the two countries even though the direction of flow is not apparent, neither is it known whether the exchange is legitimate. Nonetheless, it confirms the point that the circulation of falsified or substandard medicines could have serious public health implications for all the countries involved. To obtain better representative results in the two countries, it is recommended that subsequent quality evaluation surveys employ the more rigorous random sampling plan and also extend beyond the current sampling sites.

### 4.3. Monotherapy Medicines

Due to the potential rapid development of parasite resistance, the use of monotherapy artemisinin-based antimalarial medicines has been discouraged, while coformulated ACTs usage has been positively encouraged. As already highlighted in previous sections, collections made during this study revealed a significant circulation of monotherapy formulations of dihydroartemisinin, artesunate, and artemether still being openly distributed and sold, especially in Togo. It is important to note, however, that the more vital parenteral artesunate recommended for severe malaria was not available in any of the sampling sites.

### 4.4. The Quality of Antimalarial Medicines in Africa in Recent Years

The results obtained in this study are in consonance with other results published in recent times. Osei-Safo et al., [11] in a study on antimalarials distributed in the Accra metropolis, found that out of 23 artesunate containing samples analysed, only 3 artesunate single-dose and 1 artesunate-amodiaquine co-formulations were compliant with Ph. Int. requirements for API content. None of the six (6) artemether containing medicines passed, while one of the two dihydroartemisinin assayed by HPLC passed. Overall, the passing rate was 4 out of 31 samples analysed by HPLC, with two other samples being borderline compliant. In another study, Ofori-Kwakye et al. [8]. also reported that the artesunate content of tablets sampled in Kumasi varied between 47.9% and 99.9% of the manufacturer's label claim and only 3 (17.6%) of the samples met the European Pharmacopoeial requirements for content of active ingredients.

A more recent Survey of the quality of selected antimalarial medicines circulating in six countries of sub-Saharan Africa (Cameroon, Ethiopia, Ghana, Kenya, Nigeria and the United Republic of Tanzania) carried out by the WHO [12] found that although there was also a substantial amount of unregistered products especially among the ACTs, the nature of failure rates for registered and unregistered medicines was similar. The failure rate in terms of country comparison showed that products from Nigeria had the highest (64%), followed by Ghana (39.5%) and then Cameroon (37%).

## 5. Conclusion

The findings of the study suggest the existence of substandard artemisinin-based antimalarial medicines in both Ghana and Togo. The presence of insufficient active pharmaceutical ingredient was identified as the main cause of the poor quality. In most cases, the lacking component was the artemisinin-type medicine while the cheaper nonartemisinin component was present in sufficient quantities. The study has revealed that the registration status of antimalarial medicines on the Ghanaian market has not improved since the 2011 publication of the WHO QAMSA report on the quality of antimalarials in selected African countries including Ghana. Togo has a better registration status and cross-border activity between the two countries may not be a common phenomenon. The results also show that the registration status as well as the manufacturing source of the antimalarial medicines sampled did not have any significant impact on their quality since failure rates were comparable. This suggests that there exist inconsistencies in implementation of GMPs in both domestic and foreign products. We realise the enormity of the task and recommend that relevant departments within our universities are strengthened and accredited to assist the NMRAs undertake regular quality assurance and pharmacovigilance. We also recommend a greater enforcement of adherence to medicine registration procedures by regulators to improve the implementation of GMPs by domestic manufacturers and ensure that imported medicines are tested in WHO prequalified laboratories. In this regard, any medicine donations must be accepted only if they comply with established guidelines. Furthermore, better cooperation among all stakeholders-manufacturers, importers/exporters, distributors, regulators, and indeed the consumer must be promoted through frequent education and training.

Results obtained from the SQ-TLC assays were generally confirmed by the HPLC assays, affording another opportunity to apply and verify the suitability of the semiquantitative TLC assay as a rapid analytical tool for antimalarial medicines. One major objective achieved in the present study which the previous work did not address due to technical reasons was to develop suitable HPLC methods for concurrent assay of both components of the single tablet coformulated ACTs in the determination of their quality. In the previous study, failure or otherwise of the samples was based on only the pharmacopoeia compliance of the artemisinin component of the medicine formulation, even for ACTs which were presented or marketed as coblistered formulations.

The WHO, in its global plan for artemisinin resistance containment [41], has indicated that the effort at containing resistance to the ACTs should include among other activities the withdrawal of orally administered artemisinin-based monotherapies, substandard, and falsified medicines. The artemisinins are the chief components of the ACTs and every effort has to be made to prolong their useful therapeutic lives together with those of their partner drugs [42].

## Figures and Tables

**Table 1:** Distribution of antimalarial medicines collected from the different sampling sites.

Total products	Product type		Outlet		Zones∗						
	ACT	Monotherapy	Private	Informal	1	2	3	4	5	6	7
Ghana											
58 100%	49 84.5%	9 15.5%	49 84.5%	9 15.5%	—	6 10.3%	24 41.4%	6 10.3%	10 17.2%	10 17.2%	2 3.5%

Togo											
74 100%	41 55.4%	33 44.6%	50 67.6%	24 32.4%	38 51.4%	12 16.2%	12 16.2%	12 16.2%	—	—	—

^*^Ghana: 2: Winneba and Awutu; 3: Takoradi; 4: Bolgatanga and Sandema; 5: Aflao; 6: Kumasi; 7: Half Assini.

^*^Togo: 1: Lomé; 2: Aného; 3: Atakpame; 4: Dapaong.

**Table 2:** Categories of antimalarial medicines.

Category	Number of samples per country		
	Ghana	Togo	Subtotal
ACT formulations			
Artemether/lumefantrine coformulated	30	28	58
Artemether/lumefantrine coblistered	1	—	1
Artesunate/amodiaquine coformulated	3	5	8
Artesunate/amodiaquine coblistered	11	6	17
Dihydroartemisinin/piperaquine coformulated	4	1	6
Dihydroartemisinin/sulfadoxine/pyrimethamine coblistered	—	1	1

	49	41	90 (68.2%)

Artemisinin-based monotherapy formulations			
Artemether (parenteral)	1	21	22
Artesunate (oral)	3	11	14
Dihydroartemisinin (oral)	5	1	6

	9	33	42 (31.8%)

**Table 3:** Percentage composition and milligram quantities of active pharmaceutical ingredient (API) of a selection of samples from Ghana and Togo by SQ-TLC and HPLC and comparison of results with the stated manufacturer's claim and pharmacopoeia requirements.

Code	Manufacturer's label claim	Semiquantitative TLC estimation of composition in % and mg quantities of dosage forms of the **API** (**in bold**) compared to the manufacturer's label claim range of dosage forms (*n* = 6)				Remarks based on SQ-TLC results	HPLC determination of composition of API in % and mg quantities (*n* = 6)		Remarks based on HPLC results
		Solvent system 1		Solvent system 2				
		% range ± rsd	Quantity (mg)	% range ± rsd	Quantity (mg)		% ± rsd	Quantity (mg)
Samples from Ghana									
3N_1_	**ATS**/AMQ: **100**/270 mg	70–75 ± 0	70–75	70–75 ± 0	70–75	**NC**	70.62 ± 2	70.62	NC
3N_1_	ATS/**AMQ**: 100/**270** mg	81.0–86.0 ± 2	218.7–232.2	82.5–87.5 ± 3	222.5–236.3	**NC**	95.55 ± 0.96	257.99	C
3N_7_	**ATS**: **50** mg oral	Not detected	Not detected	Not detected	Not detected	**NC**	Not detected	Not detected	NC
4N_2_^f^	**ATS**/AMQ: **100**/270 mg coformulated	77.5–82.5 ± 3	77.5–82.5	76–81 ± 2	76–81	**NC**	83.46 ± 1	83.46	NC
4N_2_^f^	ATS/**AMQ**: 100/**270** mg coformulated	101.5–106.5 ± 2	274.1–287.6	100.5–106.5 ± 2	274.1–287.6	**C**	101.77 ± 2	274.8	C
^∧^5P_3_^f^	**ATM**/LUM: **80**/480 mg coformulated	89–94 ± 3	71.2–75.2	88–93 ± 2	70.4–74.4	**C**	96.8 ± 2	77.44	C
^∧^5P_3_^f^	ATM/**LUM**: 80/**480** mg coformulated	86–91 ± 2	412.8–436.8	88–93 ± 3	422.6–446.4	**C**	93.91 ± 4	450.77	C
5P_1_	**ATM 80** mg/1 mL injection	87.5–92.5 ± 0	70–74	90–95 ± 0	72–76	**BLC**	94.04 ± 3	75.23	BLC
6Q_1_^*^	**DHA**: **60** mg	70–75 ± 0	42–45	68–73 ± 4	40.8–43.8	**NC**	80.75 ± 2	48.45	NC
6Q_3_^f^	**DHA**/PPQ: **40**/320 mg coformulated	81–86 ± 2	32.4–34.4	84–89 ± 2	33.6–35.6	**BLC**	85.69 ± 1	34.28	NC

Samples from Togo									
1N11	**ATS**: **50 mg** oral	60–70 ± 6	30–35	60–70 ± 6	30–35	**NC**	67.07 ± 2.4	33.54	NC
3N8	**ATS**: **50 mg** oral	55–65 ± 6	27.5–32.5	54–64 ± 6	27–32	**NC**	60.34 ± 1.06	30.17	NC
1M3	**ATS**/AMQ: **100**/270 mg	67–77 ± 6	67–77	70–80 ± 5	70–80	**NC**	71.92 ± 2.07	71.92	NC
1M3	ATS/**AMQ**: 100/**270 mg**	90–100 ± 4	243–270	86.5–96.5 ± 4	233.55–260.55	**C**	95.2 ± 0.86	257.04	C
1R16	**ATM**: **80 mg/1 mL **injection	74–84 ± 6	59.2–67.2	70–80 ± 14	56–64	**NC**	93.14 ± 2.06	74.51	BLC
1RM5	**ATM**: **80 mg/1 mL **injection	90–100 ± 0	72–80	85–95 ± 8	68–76	**BLC**	88.33 ± 2.39	70.66	NC
^∧^1PM2	**ATM/**LUM**: 80**/480 mg coformulated	65–75 ± 0	52–60	70–80 ± 10	56–64	**NC**	71.51 ± 1.60	57.21	**NC**
^∧^1PM2	ATM/**LUM**: 80/**480 mg **coformulated	<60 ± 0	<288	<60 ± 0	<288	**NC**	104.22 ± 2.29	500.256	**C**
1Q21	**DHA/**PPQ**: 40 mg**/320 mg coformulated	110–117 ± 4	44–46.8	113–120 ± 8	45.2–48	**NC**	124.59 ± 1.64	49.84	NC
1QM6∗	**DHA**: **60 mg**	110–115 ± 0	66–69	110–115 ± 0	66–69	**BLC**	106.77 ± 3.39	64.06	C
1Q22#	DHA/**SDX/PYR**: 60 mg**/500 mg/25 mg** coformulated	**110–120 ± 0**	**66–72**	**107–117 ± 4**	**64.2–70.2**	NC	—	—	—

C: compliant; NC: noncompliant; BLC: borderline compliant; 6Q_1_^*^ has the same batch number as 1QM6∗; ^∧^5P_3_^f^ has the same batch number as ^∧^1PM2.

1Q22# could not be assayed by HPLC due to interference by the sulfadoxine (SDX) component.

**Table 4:** Quality of the categories of antimalarial medicines by SQ-TLC assay.

Categories of antimalarials	Ghana				Togo				Overall failure rate
	Total	Number tested	Fail	% failure	Total	Number tested	Fail	% failure	
ACTs (90)									
Artesunate	14	12	12	**100%**	11	11	11	**100%**	
Artemether	31	30	25	**83.3%**	28	28	28	**100%**	80/87 = **92.0%**
Dihydroartemisinin	4	4	2	**50%**	2	2	2	**100%**	
Artemisinin-based monotherapy (42)									
Artesunate (oral)	3	3	3	**100%**	11	11	11	**100%**	
Artemether (parenteral)	1	1	0	**0%**	21	17	14	**82.4%**	32/38 = **84.2%**
Dihydroartemisinin (oral)	5	5	4	**80%**	1	1	0	**0%**	

**Table 5:** Quality of the categories of antimalarial medicines by HPLC assay.

Categories of antimalarials	Ghana				Togo				Overall failure rate
	Total	Number tested	Fail	% failure	Total	Number tested	Fail	% failure	
ACTs (90)									
Artesunate	14	12	12	**100%**	11	11	11	**100%**	
Artemether	31	30	22	**73.3%**	28	28	24	**85.7%**	72/86 = **83.7%**
Dihydroartemisinin	4	4	2	**50%**	2	1	1	**100%**	
Artemisinin-based monotherapy (42)									
Artesunate (oral)	3	3	3	**100%**	11	11	11	**100%**	
Artemether (parenteral)	1	1	0	**0%**	21	17	5	**29.4%**	22/38 = **57.9%**
Dihydroartemisinin (oral)	5	5	3	**60%**	1	1	0	**0%**	

**Table 6:** Quality of antimalarial medicines versus manufacturing source and registration status.

Country	Number of samples	Manufacturing source						Registration status					
		Domestic			Foreign			Registered			Unregistered		
		Total	Number tested	Fail	Total	Number tested	Fail	Total	Number tested	Fail	Total	Number tested	Fail
Ghana	58	14	13	12	44	42	32	13	12	10	45	43	34
% failure				**92.3**			**76.2**			**83.8**			**79.1**
Togo	74	9	9	5	65	60	47	58	53	36	16	16	16
% failure				**55.6**			**78.3**			**68.0**			**100**

Total	132	23	22	17	109	102	79	71	65	46	61	59	50
Overall % failure				**77.3**			**77.5**			**70.8**			**84.7**
